# Soluble CD40 Ligand and Oxidative Response Are Reciprocally Stimulated during Shiga Toxin-Associated Hemolytic Uremic Syndrome

**DOI:** 10.3390/toxins9110331

**Published:** 2017-10-25

**Authors:** Maria J. Abrey Recalde, Romina S. Alvarez, Fabiana Alberto, Maria P. Mejias, Maria V. Ramos, Romina J. Fernandez Brando, Andrea C. Bruballa, Ramon A. Exeni, Laura Alconcher, Cristina A. Ibarra, María M. Amaral, Marina S. Palermo

**Affiliations:** 1 Laboratorio de Patogénesis e Inmunología de Procesos Infecciosos, Instituto de Medicina Experimental, Consejo Nacional de Investigaciones Científicas y Técnicas-Academia Nacional de Medicina, 1425 Buenos Aires, Argentina; jimena.abrey@gmail.com (M.J.A.R.); mpmejias@gmail.com (M.P.M); vramos@hematologia.anm.edu.ar (M.V.R.); rominbra@yahoo.com.ar (R.J.F.B.); andrea.bruballa@gmail.com (A.C.B.); 2 Laboratorio de Fisiopatogenia, Departamento de Fisiología, Instituto de Fisiología y Biofísica “Bernardo Houssay”, Facultad de Medicina-Consejo Nacional de Investigaciones Científicas y Técnicas, Universidad de Buenos Aires, 1121 Buenos Aires, Argentina; roalvarez_4@yahoo.com.ar (R.S.A.); cristinaadrianaibarra@gmail.com (C.A.I); mmamaral74@gmail.com (M.M.A.); 3 División Trombosis, Instituto de investigaciones Hematológicas “Mariano R. Castex”, Academia Nacional de Medicina, 1425 Buenos Aires, Argentina; fabiana@hematologia.anm.edu.ar; 4 Departamento de Nefrología, Hospital Municipal del Niño, San Justo, B1754FUD Provincia de Buenos Aires, Argentina; Raexeni@gmail.com; 5 Unidad de Nefrourología Infantil. Hospital Interzonal General Dr. José Penna, Bahía Blanca, 8000 Provincia de Buenos Aires, Argentina; Laura.alconcher.la@gmail.com

**Keywords:** hemolytic uremic syndrome, oxidative stress, blood platelets, Shiga toxin 2, CD40L

## Abstract

Shiga toxin (Stx), produced by *Escherichia coli*, is the main pathogenic factor of diarrhea-associated hemolytic uremic syndrome (HUS), which is characterized by the obstruction of renal microvasculature by platelet-fibrin thrombi. It is well known that the oxidative imbalance generated by Stx induces platelet activation, contributing to thrombus formation. Moreover, activated platelets release soluble CD40 ligand (sCD40L), which in turn contributes to oxidative imbalance, triggering the release of reactive oxidative species (ROS) on various cellular types. The aim of this work was to determine if the interaction between the oxidative response and platelet-derived sCD40L, as consequence of Stx-induced endothelium damage, participates in the pathogenic mechanism during HUS. Activated human glomerular endothelial cells (HGEC) by Stx2 induced platelets to adhere to them. Although platelet adhesion did not contribute to endothelial damage, high levels of sCD40L were released to the medium. The release of sCD40L by activated platelets was inhibited by antioxidant treatment. Furthermore, we found increased levels of sCD40L in plasma from HUS patients, which were also able to trigger the respiratory burst in monocytes in a sCD40L-dependent manner. Thus, we concluded that platelet-derived sCD40L and the oxidative response are reciprocally stimulated during Stx2-associated HUS. This process may contribute to the evolution of glomerular occlusion and the microangiopathic lesions.

## 1. Introduction

Shiga toxin (Stx)-producing *Escherichia coli* (STEC) is associated with the development of hemolytic uremic syndrome (HUS), which is characterized by hemorrhagic diarrhea followed by microangiopathic hemolytic anemia, thrombocytopenia, and acute renal failure [1].

After ingestion, STEC colonize the intestine and produce Stx that translocates across the intestinal epithelium [2]. Stx is considered as the main pathogenic factor and necessary for HUS development [3]. Once Stx enters into bloodstream, it binds to its specific globotriaosylceramide (Gb3) receptor, which is present on microvascular endothelial cells and epithelial cells of target organs and monocytes. The main target organ is the kidney, but other organs such as the brain are also affected [4].

Endothelial damage plays a crucial role in the sequence of events leading to the microangiopathic process during HUS [5]. Microvascular endothelial cells, mostly of renal glomeruli, become activated in response to Stx and lose their anti-thrombogenic properties. This leads to the upregulation of adhesive molecules, such as vitronectin receptor, PECAM-1, and P-selectin on endothelial surface that mediate platelet adhesion and thrombi formation [6]. Thus, platelet adhesion and subsequent aggregation contribute to the formation of platelet-fibrin thrombi characteristic of the thrombotic microangiopathy during Stx-associated HUS [7].

Furthermore, soluble factors such as cytokines and/or chemokines released by Stx-activated microvascular endothelium [8] and monocytes [9] have also been implicated in platelet activation. Thereby, thrombosis and inflammation are strictly correlated and constitute the major pathogenic components of Stx-associated HUS.

Studies performed on platelets from patients with HUS showed impaired aggregating responses [10,11] and reduced β-thromboglobulin content [11], indicating that the aggregation process had occurred in vivo. However, aggregation is not the only function of activated platelets. It has been recently recognized that platelets modulate immuno-inflammatory reactions through cytokine secretion and subsequent interaction with leukocytes and endothelial cells [12]. In fact, platelets are an important source of potent autocrine and paracrine factors, including several vasoactive and inflammatory mediators, such as P-selectin, CD40 ligand (CD40L), chemokines, growth factors, and others [13,14]. CD40L, a membrane glycoprotein belonging to the TNF superfamily, is expressed mainly by activated CD4-T cells and activated platelets [15,16]. Platelet CD40L is stored in α-granules and is translocated to the platelet surface upon activation [17,18]. Surface-expressed CD40L is then cleaved over a period of minutes to hours, generating a soluble fragment, soluble CD40L (sCD40L). It remains trimeric, so retaining the ability to activate its widely expressed receptor CD40 [15], promoting inflammatory or thrombotic response by causing further platelet activation [17]. More than 95% of circulating CD40L is derived from platelets [19,20].

CD40-CD40L interaction is decisive to induce B-cell proliferation, to generate memory B cells, and mediates antibody class switching. However, it was subsequently shown that CD40L and CD40 are also present on several cells of the vasculature, including endothelial cells, smooth muscle cells, monocytes, and platelets. Platelet-associated CD40L is capable of initiating various inflammatory responses, including expression of inflammatory adhesion receptors, expression of tissue factor, and release of chemokines and cytokines (e.g., monocyte chemoattractant protein-1 [MCP-1], interleukin-6, and interleukin-8) [21]. In this regard, sCD40L has been implicated in the pathogenesis of atherosclerosis and other immuno-inflammatory diseases [22,23,24,25,26,27,28].

The biological function of sCD40L has recently been a subject of intense investigation; sCD40L binds to CD40 on target cells triggering an inflammatory response [18,22,29,30]. Furthermore, it is capable to induce oxidative stress and reactive oxidative species (ROS) generation in various cellular types such as endothelial cells [26], monocytes [31,32], platelets [33], and neutrophils [34]. The involvement of the oxidative stress in tissue damage and renal failure processes during Stx-intoxication has also been subject of investigation; oxidative stress is generated by Stx systemically and locally in the kidney and has been shown to enhance platelet activation [35]. Thus, oxidative stress and platelet-derived sCD40L could stimulate each other.

In the present study, we investigated the effects of Stx2 and oxidative stress on renal microvasculature, platelet adhesion, and sCD40L release in order to identify a novel mechanism contributing to thrombotic microangiopathy. The in vivo release of sCD40L and its role in oxidative stress was demonstrated in HUS patients.

## 2. Results

### 2.1. Platelets Did Not Contribute to Stx2-Mediated Damage to Human Glomerular Endothelial Cells (HGEC)

In order to study the interplay between renal endothelium and platelet activation when Stx is present, confluent HGEC cultures were incubated overnight with different concentrations of purified Stx2. Then, isolated human platelets (1 × 10^8^) were added or not to HGEC cultures. After 1 h, HGEC were stained with H&E and observed by optical microscopy to register any change in the cellular morphology associated with toxicity (shape and cellular detachment). In parallel, the viability of living cells was analyzed by the neutral red assay.

**Figure 1:**
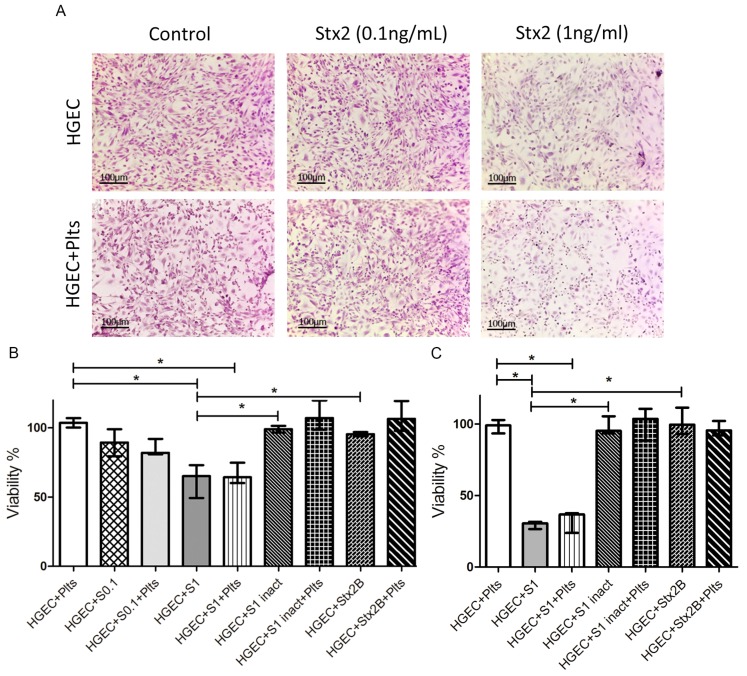
Microvasculature damage by Stx2. Human glomerular endothelial cells (HGEC) were seeded on gelatin-coated glass coverslips (**A**), or 24-well plates (**B**,**C**) treated or not with 0.1 ng/mL (S0.1), 1 ng/mL (S1) Stx2, 1 ng/mL Stx2 inactivated by heating (S1 inact) or 1 ng/mL Stx2B (Stx2B). After 24 h, platelets (Plts) (1 × 108/well) were added at 37 °C in 5% CO_2_ for 1 h and washed. (**A**) Representative images by light microscopy from each experimental condition stained with H&E (×10) are shown. Viability was evaluated 1 h (**B**) or 18 h (**C**) after Plts addition. Cells were incubated with neutral red an additional hour at 37 °C in 5% CO_2_. The percentage of viable cells was calculated considering that absorbance (**A**) obtained for cells incubated without toxin treatment represents 100% viability. Each condition was made in duplicate for each experiment. Data are expressed as median and interquartile range of three independent experiments (*n* = 3). * *p* < 0.05.

Although no significant changes were observed with 0.1 ng/mL Stx2, 1 ng/mL Stx2 induced a significant toxicity on HGEC as it was demonstrated by a decreased uptake of the vital dye neutral red (Figure 1A,B). We also evaluated the effect of heat-inactivated Stx2 (S1 inact) and the isolated B subunit of Stx2 (Stx2B) to confirm that enzymatic activity is required to damage the endothelium. No additional toxicity signs were evident after one hour (Figure 1B) or 18 h (Figure 1C) from platelet addition. These results confirmed the dose-dependent toxic effect of Stx2 on HGEC, and showed that platelets did not enhance endothelial damage, even when HGEC were incubated with Stx2 at a suboptimal concentration.

### 2.2. Stx2-Mediated Damage to HGEC Induced Platelet Adhesion

**Figure 2:**
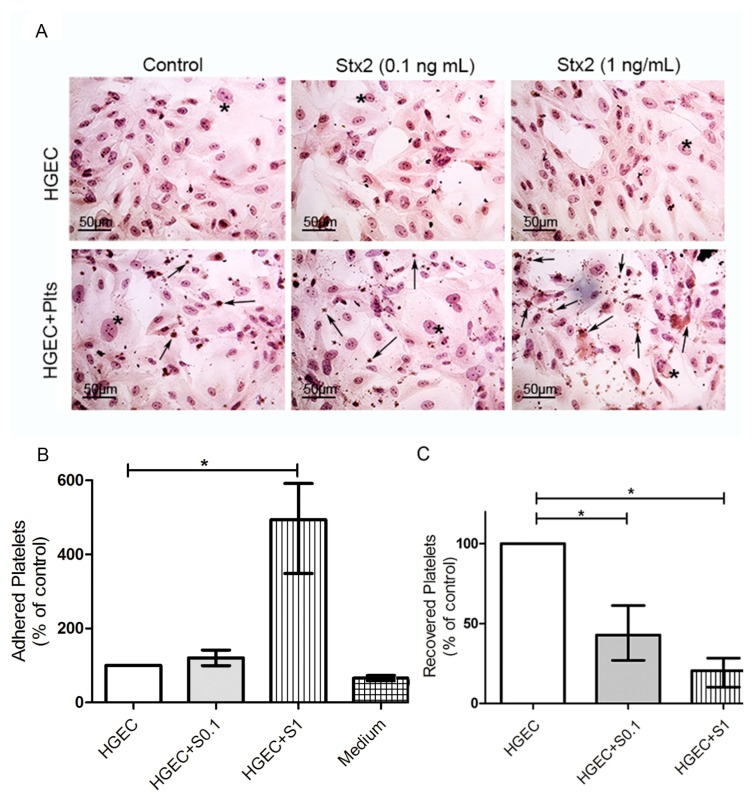
Platelet adhesion to damaged endothelium. HGEC were seeded on gelatin-coated glass coverslips (**A**), or 24-well plates (**B**,**C**) treated or not with 0.1 ng/mL (S0.1) or 1 ng/mL (S1) Stx2. After 24 h, Plts (1 × 10^8^/well) were added for 1 h at 37 °C in 5% CO_2_ and washed. (**A**) Representative images by light microscopy from each experimental condition stained with H&E (×400) are shown. HGEC are indicated by asterisk and Platelets by arrows. (**B**) Cells were incubated with PNP and acid phosphatase activity was measured reading *A* at 405 nm. *A* obtained in wells with HGEC without platelets was subtracted from that obtained in wells with platelets for each experimental condition. The percentage of adhered platelets was calculated considering that wells without Stx2 treatment represents 100%. The control of platelet adhesion to gelatin-covered well is shown as medium. (**C**) Supernatants from HGEC-Plts cultures were collected and the number of Plts recovered was determined by hematology analyzer. The number of Plts recovered in HGEC cultures without Stx2 treatment represented 100%. Each condition was made in duplicate for each experiment. Data are expressed as median and interquartile range of five independent experiments (*n* = 5). * *p* < 0.05.

Then, we evaluated whether platelets respond to Stx2-mediated endothelial injury by increasing their adhesion. Platelet adhesion was measured by the acid phosphatase assay, reading *A* at 405 nm and subtracting the *A* obtained for each experimental condition without platelets, because all living cells (i.e., HGEC) show acid phosphatase activity. Figure 2B shows a significant increase in the percentage of adhered platelets only when they were added to HGEC cultures that were pre-treated with 1 ng/mL Stx2, indicating that the damaged endothelium stimulated platelet adhesion. This conclusion was confirmed by counting the platelets recovered from cultures under different experimental conditions. The percentage of platelets recovered was around 50% from HGEC pre-treated with 0.1 ng/mL Stx2 and only 25% from HGEC pre-treated with 1 ng/mL Stx2 compared to platelets recovered from non-treated HGEC (Figure 2C). These results suggest that although Stx2-induced endothelial injury was not evident, platelet adhesion was triggered even at sub-optimal Stx2 doses. However, maximal platelet adhesion was observed when HGEC were incubated with 1 ng/mL Stx2.

### 2.3. Stx2-Mediated HGEC Damage Induced Platelet to Release sCD40L

Then, we further analyzed if platelets, besides showing an increased adhesion to damaged endothelium, were able to release sCD40L upon incubation with Stx2-damaged HGEC. For this purpose, culture supernatants under the same experimental conditions were recovered and sCD40L was quantified by ELISA with a commercial kit.

**Figure 3:**
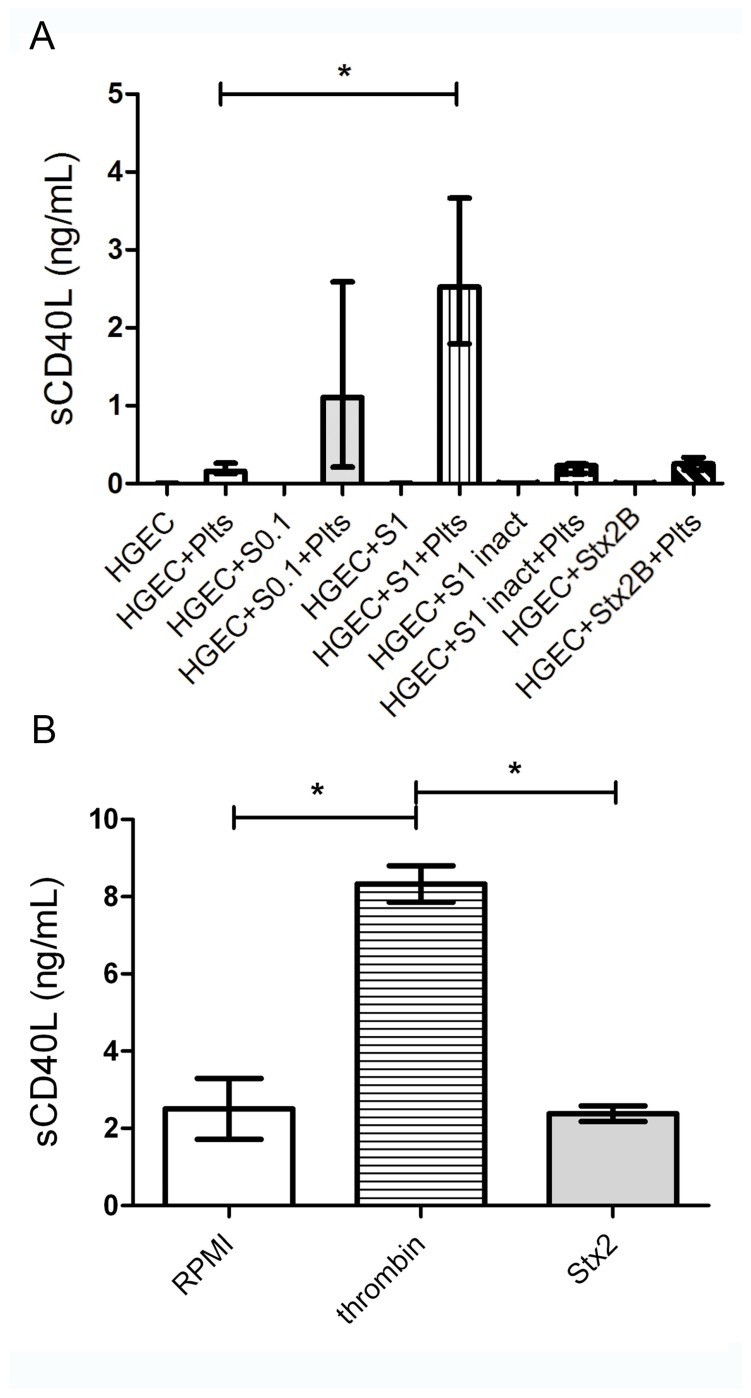
Release of sCD40L by Plts. (**A**) HGEC were placed in gelatin-covered 24-well plates treated or not with 0.1 ng/mL (S0.1), 1 ng/mL (S1) Stx2, 1 ng/mL Stx2 inactivated by heating (S1 inact) or 1 ng/mL Stx2B. After 24 h, Plts (1 × 10^8^/well) were added for 1 h at 37 °C in 5% CO_2_ and washed. Supernatants of HGEC cultures were collected and sCD40L levels were measured by ELISA kit. (**B**) Isolated Plts (1 × 10^8^/well) were stimulated with 0.2 U/mL thrombin or 1 ng/mL Stx2 during 1 h at 37 °C in 5% CO_2_ and centrifuged. Supernatants were collected and sCD40L levels measured by ELISA kit. Each condition was made in duplicate for each experiment. Data are expressed as median and interquartile range of eight independent experiments (*n* = 8). * *p* < 0.05. Controls with S1 inact and Stx2B were performed 3 times and each condition was made by duplicate.

As shown in Figure 3A, supernatants from platelets incubated with 1 ng/mL Stx2-treated HGEC, had significant increased levels of sCD40L. In contrast, supernatants from platelets incubated with S1inact or Stx2B-treated HGEC, did not show sCD40L, suggesting that Stx2-damaged HGEC were able to induce platelets to release this pro-inflammatory mediator. As expected, HGEC pre-treated with 1 ng/mL Stx2, did not produce sCD40L, confirming that platelets are the major source of sCD40L. Moreover, HGEC incubated with a suboptimal Stx2 dose, stimulated a low, but not significant, release of sCD40L by platelets.

When considering that controversial results regarding Stx direct effect on platelets have been shown [36,37,38,39], we determined if Stx2 was able to directly stimulate platelets to release sCD40L. Thus, platelets were incubated with 1 ng/mL Stx2 for one hour and thrombin was used as a positive control. Then, supernatants were collected and sCD40L concentration measured by ELISA.

As shown in Figure 3B, supernatants from Stx2-stimulated platelets have a similar sCD40L concentration than control platelets incubated with medium. In contrast, supernatants from thrombin-stimulated platelets presented a significant increase in sCD40L levels. It is important to highlight that although Stx2 was not removed from the HGEC cultures before adding platelets, supernatants were tested on VERO cells and did not show toxic activity (data not shown).

These results suggest that Stx2 did not directly stimulate platelets to release sCD40L, but instead, indirectly through endothelial damage.

### 2.4. ROS Did Not Contribute to Stx2-Mediated Damage to HGEC or Platelet Adhesion

Stx2 intoxication induced a marked prothrombotic status and simultaneously a pro-oxidative imbalance, demonstrated in both experimental mouse models [35], and most important in HUS-patients [40,41]. Considering that N-acetylcysteine (NAC) is a well-known anti-oxidant and glutathione precursor [42], we decided to evaluate if it was able to inhibit Stx2-mediated effects on HGEC cultures.

HGEC incubated with or without 1 ng/mL Stx2 were incubated with or without 1mM NAC [34] and toxicity and viability were analyzed by H&E staining and neutral red uptake, respectively.

**Figure 4:**
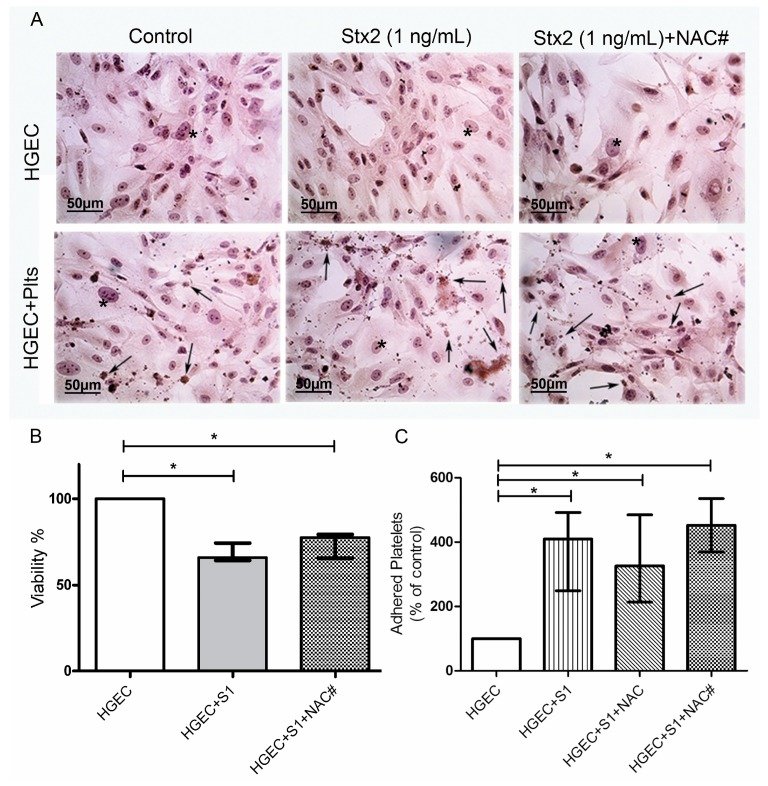
Role of oxidative stress in endothelial damage and platelet adhesion. HGEC were seeded on gelatin-covered glass coverslips (**A**) or 24-well plates (**B**,**C**) treated or not with 1 ng/mL (S1) Stx2. After 24 h, Plts (1 × 10^8^/well) were added for 1 h at 37 °C in 5% CO_2_. NAC (1 nM) was incorporated simultaneously with Stx2 (NAC#) or previous to Plts (NAC). (**A**) Representative images by light microscopy from each experimental condition stained with H&E (×400). Number of viable cells and Plts adhered to HGEC were observed by light microscopy (×400). HGEC are indicated by asterisk and Platelets by arrows; (**B**) Cells were incubated with neutral red for an additional 1 h at 37 °C in 5% CO_2_. The percentage of viable cells was calculated considering that *A* obtained for cells incubated without toxin treatment represents 100% viability; (**C**) Cells were incubated with PNP and acid phosphatase activity was measured reading *A* at 405 nm. *A* obtained in wells with HGEC without platelets was subtracted from that obtained in wells with platelets. The percentage of adhered platelets was calculated considering that wells without Stx2 treatment represents 100% of adhered platelets. Each condition was made in duplicate for each experiment. Data are expressed as median and interquartile range of eight independent experiments (*n* = 8). * *p* < 0.05.

We did not observe any difference in the viability of HGEC between Stx2-treated and Stx2/NAC-treated cultures, showing that anti-oxidant treatment did not inhibit injury directly caused by Stx2 to the endothelium (Figure 4A,B). Then, we determined platelet adhesion, as previously described. We tested NAC effect in two experimental protocols, incorporating NAC from the beginning of culture (together with 1 ng/mL Stx2), and incorporating NAC previous to platelets addition. As shown in Figure 4C, NAC did not inhibit the increase of platelet adhesion, secondary to Stx2-mediated endothelial damage, in any of the protocols evaluated. These results demonstrate that ROS present in medium does not contribute to endothelial damage directly induced by Stx2. Moreover, antioxidant treatment was not able to significantly inhibit platelet adhesion induced by Stx2-damaged endothelium.

### 2.5. Oxidative Stress was Involved in the Platelet sCD40L Release

**Figure 5:**
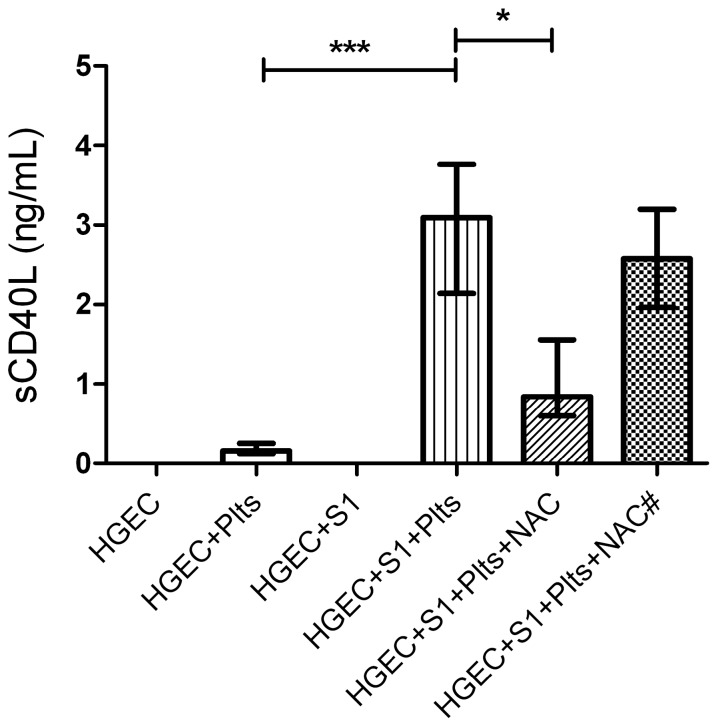
Role of oxidative stress in sCD40L platelet release. HGEC were placed in 24-well plates treated or not with 1 ng/mL (S1) Stx2. After 24 h Plts (1 × 10^8^/well) were added for 1 h at 37 °C in 5% CO_2_. NAC (1 nM) was incorporated simultaneously with Stx2 (NAC#) or previous to Plts (NAC). Supernatants of HGEC cultures were collected and sCD40L levels were measured by ELISA kit. Each condition was made in duplicate for each experiment. Data are expressed as median and interquartile range of eight independent experiments (*n* = 8). * *p* < 0.05, *** *p*< 0.001.

Even though NAC did not modify Stx2-mediated endothelial damage or platelet adhesion, we further analyzed whether NAC affected the release of sCD40L by platelets under the same experimental protocol. Supernatants were collected after one hour of platelet addition, and sCD40L was quantified by ELISA, as previously described. Figure 5 shows that supernatants from platelets incubated with Stx2-pretreated HGEC had increased sCD40L levels, and sCD40L release was significantly inhibited only when NAC was added before platelets. The fact that NAC was effective on blocking sCD40L release but not platelet adhesion, suggests that both processes are modulated by different pathways and oxidative stress is only involved in platelet degranulation.

### 2.6. HUS Patients Had Increased Levels of sCD40L in Circulation

**Figure 6:**
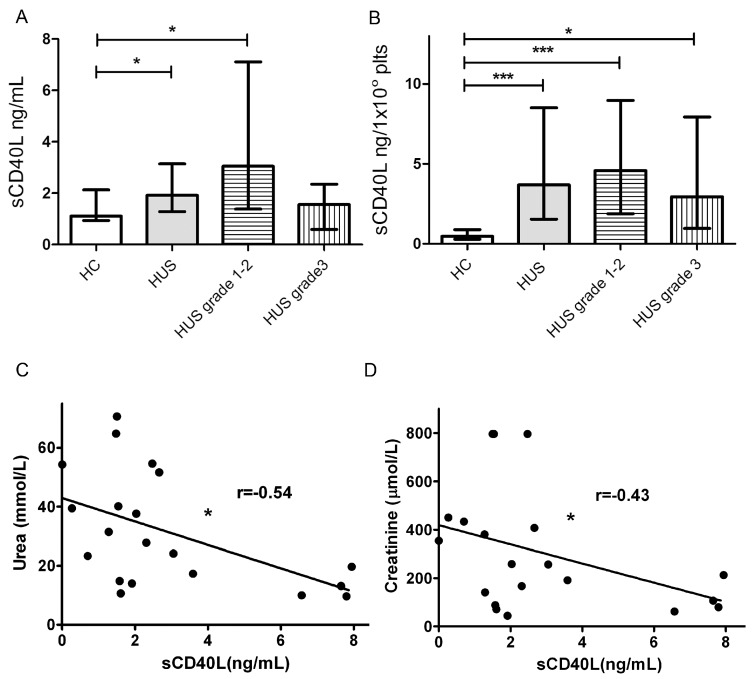
sCD40L levels in hemolytic uremic syndrome (HUS) plasma. (**A**) sCD40L levels in HUS patients and healthy controls (HC) plasmas were determined by ELISA kit. In addition, HUS patients were retrospectively classified according to Gianantonio et al.’s criteria in grade 1/2 (*n* = 13) and grade 3 (*n* = 10). Data are expressed as median and interquartile range; (**B**) Quantities of sCD40L (ng) released per 1 × 10^8^ Plts from HUS patients and HC. Data are expressed as median and interquartile range; (**C**) Correlation between sCD40L and plama creatinine levels in HUS patients; (**D**) Correlation between sCD40L and plasma urea levels in HUS patients. Points represent independent individuals. * *p* < 0.05 *** *p* < 0.001.

Because we found that Stx2-mediated damage to HGEC leads to the release of sCD40L by platelets, we assayed the plasma levels of sCD40L in HUS patients and healthy controls (HC). With this aim, we collected plasma from HUS patients at diagnosis and healthy age-matched controls, and sCD40L concentration was quantified by ELISA. In addition, HUS patients were retrospectively classified according their evolution and renal dysfunction. Thus, patients were classified in two groups: those classified as grade 1 and 2 (up to 7 days of dialysis) and grade 3 (more than 7 days of anuria or dyalisis), according to Gianantonio et al.’s criteria [43]. As depicted in Figure 6A, HUS patients had increased levels of sCD40L in circulation. Interestingly, when HUS were separated according severity score, only plasmas from grade1–2 patients had significantly increased sCD40L levels, as compared to HC, while grade 3 patients presented sCD40L circulating levels similar to controls. Considering that platelets are the major source of sCD40L and HUS patients had different degree of thrombocytopenia, we analyzed the relationship between the number of circulating platelets and the plasmatic levels of sCD40L in HUS patients and HC. Figure 6B shows that HUS patients from all of the severity groups had higher values of sCD40L produced per platelet than HC, indicating that platelets are activated and releasing sCD40L.

Finally, we analyzed the existence of any correlation between levels of sCD40L and renal dysfunction in HUS patients, assessed as plasma levels of urea and creatinine (Figure 6C,D). We found a weak negative correlation between sCD40L and urea and creatinine levels.

All together, these results suggest that platelets of all severity groups release sCD40L, confirming their activated state. Moreover, high levels of circulating sCD40L at HUS diagnosis, could correlate with a preserved renal function.

### 2.7. Plasma sCD40L Induced ROS Generation by Monocytes

**Figure 7:**
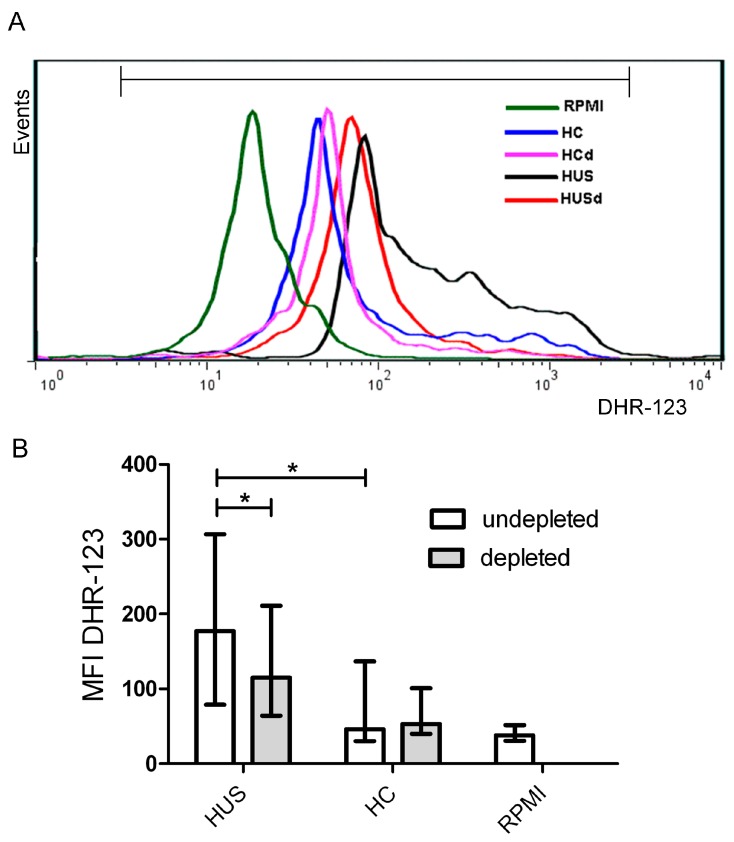
Reactive oxidative species (ROS) production by monocytes. Peripheral blood mononuclear cells (PBMC) were incubated 1h with plasmas from HUS patients or HC. Then, PMBC were washed and incubated with DHR-123 and ROS production by monocytes was measured by flow cytometry. Monocytic population was gated by FSC-H/SSC-H and CD14 staining. (**A**) Representative histogram of one experiment: PBMC incubated with: HC (healthy control plasma), HCd (Healthy control plasma depleted of sCD40L), HUS (HUS plasma) and HUSd (HUS plasma depleted of sCD40L; (**B**) Each bar shows the mean fluorescence intensity (MFI) of monocytes from 6 independent donors under different conditions. Data are expressed as median and interquartile range. * *p* < 0.05.

One of the consequences of CD40-CD40L interaction is the generation of ROS by endothelial cells [26] or monocytes [31,32]. Thus, we further examined whether plasmas from patients and controls induced ROS generation by monocytes and if this process was related to the concentration of sCD40L. For this, PBMC were incubated with plasma from HUS patients containing high levels of sCD40L (>3 ng/mL) or plasma from HC (≈1.5 ng/mL) during one hour and respiratory burst was evaluated by flow cytometry using DHR-123 as substrate. ROS generation in monocytes was evaluated analyzing MFI in monocyte gate. Plasma samples from HUS patients induced a higher ROS generation by monocytes as compared to plasma samples from HC (Figure 7B). In order to determine the role of plasma sCD40L in triggering ROS generation, it was immunodepleted with an anti-CD40L antibody. Plasma samples depleted or not of sCD40L, were analyzed in parallel. Figure 7B shows that sCD40L depletion significantly decreased ROS generation induced by HUS plasmas, but did not modify the ROS generation induced by control plasmas.

These results suggest that sCD40L present in plasma from HUS patients is biologically active, inducing monocytes to release ROS.

## 3. Discussion

Platelets play a significant role in the development of thrombosis and inflammation, two effector arms of the HUS pathogenesis. Particularly, it has been reported that platelets interact with monocytes via the CD40-CD40L pathway leading to the release of ROS and proinflammatory mediators [31,32,44,45] and monocyte adherence to the inflamed endothelial layer [46,47,48].

Results presented here lead us to propose that the interaction between platelets and monocytes occurs in a pathogenic loop that involves the CD40-CD40L pathway and the oxidative response. It is important to clarify that we choose to work with Stx2, because it is known that it is the Stx-variant most associated with human disease and HUS [49,50]. Furthermore, several previous studies have shown significant endothelial lethal dose 50 (LD50) differences between Stx1 and Stx2 [51,52].

The primary pathogenic event during systemic complications secondary to STEC infections is the microvascular injury mediated directly and/or indirectly by Stx. As consequence, platelets adhere to the endothelium and release sCD40L into the circulation in a redox-sensitive manner. sCD40L binds to CD40 on monocytes, triggering ROS generation. Thus, platelets and monocytes are reciprocally stimulated via sCD40L-CD40 dyad and ROS, secondary to microvascular injury. In this regard, we demonstrated that release of sCD40L by platelets was induced by Stx2-damaged endothelium and not directly by Stx2. In addition, the treatment of endothelium with antioxidant 1h before platelet addition significantly inhibited the release of sCD40L, suggesting that ROS derived from endothelial cells and/or from platelets itself, could stimulate sCD40L release. In contrast, antioxidant treatment added 24 h before platelets was not able to inhibit sCD40L release, probably as consequence of a rapid loss of antioxidant capacity of NAC and a sustained ROS production by endothelial cells and/or platelets. In this regard, previous studies have shown that platelet expression of CD40L involves activation of the NADPH oxidase subunit, gp91phox [53], and subsequent studies revealed that the release of sCD40L by platelets involves oxidative stress, and is inhibited by antioxidants, such as vitamin C [54]. It has also been reported that NAC is able to decrease sCD40L release by platelets upon thrombin stimulation [34].

In conclusion, the enhancement of sCD40L release following platelet exposure to Stx2-injured HGEC and the NAC blocking effect implies that endothelial-mediated platelet activation is potentiated by ROS.

The fact that plasma samples from HUS patients show elevated levels of sCD40L at the moment of diagnosis, suggests that the in vitro mechanism proposed in this study may take place in vivo during the acute disease. It is important to highlight that the highest levels of sCD40L were detected in plasmas from those patients classified as mild/moderate (grade 1–2). However, when the amount of sCD40L released per platelet was evaluated, it was observed that all of the groups of patients (grade 1–3) have an increased sCD40L production when compared to HC. Moreover, it is important to highlight that the levels of sCD40L locally released in the renal microvascular environment should be higher than those detected in systemic circulation.

Since we found a negative correlation between plasma sCD40L concentration and urea and creatinine levels in HUS patients, and considering that sCD40L levels per platelet were higher in HUS patients that HC, we propose that sCD40L could represent an alternative marker of platelet activation in those cases in which thrombocytopenia is not evident.

The sCD40L plasma values showed by HUS patients were similar to those reported in other pathological conditions, such as cardiovascular diseases, diabetes, HIV infection, or smokers [47,55,56,57,58,59] and it can be considered as a marker of thrombotic risk [20]. Therefore, and based on the present results, we propose that the quantification of sCD40L in plasma could be used as a surrogate marker of microvascular dysfunction and/or platelet activation in Stx-associated diarrheas.

The biological activity of circulating sCD40L in HUS patients was further confirmed by its capacity to induce the oxidative burst in monocytes. The enhancement of ROS generation by monocytes upon incubation with plasmas from HUS patients with elevated levels of sCD40L supports the notion that platelets may stimulate ROS generation via the CD40-CD40L interaction. The specificity of the CD40-CD40L pathway in this reaction was demonstrated using plasmas that were depleted of sCD40L, which induced a lower ROS production compared to the same plasmas previous to sCD40L depletion. In line with these results, Ha et al., demonstrated that CD40 ligation on monocytes enhances the production of ROS via activation of NADPH oxidase and PI-3-Kinase [60].

Thus, the sCD40L present in plasmas from HUS patients is able to interact with monocytes and to trigger ROS production. This in turn would contribute to endothelial damage and to further platelet activation, leading to a positive feedback loop between platelets and monocytes, in which ROS and sCD40L stimulates each other. This represents a new pathogenic pathway mediated by monocyte-platelet interaction during HUS, which adds to the already described monocyte-platelet aggregate formation [61].

Oxidative stress is widely considered as a common signaling mechanism of the vascular response to injury. Enhancement of the oxidative stress has been reported in patients with HUS [40,41] and vascular diseases, which have also been associated with elevated levels of plasma sCD40L, as has been discussed above [62,63,64]. In this regard, other authors have previously reported that recombinant human CD40L promotes oxidative burst in human neutrophils via a PI3-kinase-dependent signaling pathway [34,65,66] and Vanichakarn et al., described a positive feedback loop between platelets and neutrophils [34], similar to that reported in our study between platelets and monocytes.

In conclusion, we described a new pathway of platelet-monocyte interaction, mediated by sCD40L and oxidative stress that may contribute to the progression of endothelial dysfunction during Stx2-associated HUS. Moreover, we suggest that antioxidant treatments may be useful to reduce platelet activation and thrombus formation improving renal microcirculation and kidney function.

## 4. Materials and Methods

### 4.1. Reagents

Recombinant purified Stx2 was purchased from Tufts University, Boston, USA. It contained less than 5 pg of LPS (per µg of Stx) quantified by Limulus amebocyte lysate assay. When needed, Stx2 was inactivated (S1 inact) via heating for 1 h at 100 °C. B subunit of Stx2 (Stx2B) was produced as previously described [67]. N-acetylcysteine (NAC), dihydrorhodamine-123 (DHR-123), phorbol myristate acetate (PMA), prostaglandin E_1_ (PGE_1_) M199, endothelial cell growth supplement (ECGS), and thrombin were obtained from Sigma (St Louis, MO, USA). Fetal calf serum (FCS), l-glutamine, and penicillin/streptomycin was obtained from GIBCO (San Diego, CA, USA).

### 4.2. HGEC Cultures

Human glomerular endothelial cells (HGEC) were isolated from kidneys that were removed from different pediatric patients undergoing nephrectomies performed at Hospital Nacional “Alejandro Posadas”, Provincia de Buenos Aires, Argentina (written informed consent was obtained from the next of kin, caretakers, or guardians on the behalf of the children participants involved in our study). The Ethics Committee of the University of Buenos Aires approved the use of human renal tissues for research purposes. The method used for HGEC isolation was previously described [68].

### 4.3. Human Platelets Isolation

Blood samples were obtained from healthy donors. This study was performed according to institutional guidelines (Academia Nacional de Medicina, Buenos Aires, Argentina) and was approved by the Institutional Ethics Committee. Written consent was obtained from all of the subjects. Four volumes of blood were drawn directly into plastic tubes containing one volume of acid-citrate-dextrose (ACD). Platelet rich plasma (PRP) was obtained by the centrifugation of blood samples at room temperature (RT) at 200× *g* for 15 min. PRP samples were centrifuged at RT at 800× *g* for 10 min in presence of PGE_1_ (4 µg/mL). The pellet was suspended in washing buffer (0.01 M Tris, 0.15 M NaCl, pH: 7.4) and centrifuged at RT at 800× *g* for another 10 min in presence of PGE_1_. The final pellet was suspended in RPMI at 1 × 10^9^/mL.

### 4.4. HGEC-Platelets Cultures

HGEC were plated (50,000 cells/well) in gelatin coated 24-well plates and grown to confluence in complete medium (M199 medium supplemented with 20% FCS, 3.2 mM L-glutamine, 100 U/mL penicillin/streptomycin and 25 µg/mL ECGS). Cells were then exposed to Stx2 (0.1 ng/mL or 1 ng/mL) in growth-arrested conditions for 24 h. Then, platelets (1 × 10^8^/well) were incorporated for 1 h at 37 °C in 5% CO_2_. After that, supernatants were collected and stored at −20 °C. Cytotoxicity was evaluated at this point and 18 h after platelet addition.

### 4.5. NAC Treatment

NAC (1mM) was added to HGEC cultures simultaneously with Stx2 or 1 h before platelet addition.

### 4.6. Hematoxylin-Eosin (H&E) Staining

HGEC were seeded in glass coverslips and after treatments cells were fixed for 2 h at room temperature with alcohol 96°, stained with (H&E) hematoxylin-eosin, and observed by light microscopy, as previously described [68,69].

### 4.7. Neutral Red Cytotoxicity Assay

The neutral red cytotoxicity assay was adapted from previously described protocols [70]. Briefly, HGEC were incubated with five hundred microliters of freshly diluted neutral red in M199 to a final concentration of 10 μg/mL during 1 h at 37 °C in 5% CO_2_. Cells were then washed with 500 μL 1% CaCl_2_ + 1% formaldehyde and solubilized in 500 μL 1% acetic acid in 50% ethanol. Absorbance (*A*) in each well represents neutral red uptake and was read in an automated plate spectrophotometer at 540 nm. The percentage of viable cells was calculated when considering that *A* obtained for cells without toxin treatment represents 100% viability.

### 4.8. Acid Phosphatase Assay

This assay was made as previously described [71]. Briefly, after treatments, HGEC were washed twice with PBS and were then incubated with 500 µL of p-nitrophenylphosphate (PNP) diluted in reaction buffer (0.1 M sodium citrate, 0.1% Triton X-100, pH: 5.4) to a final concentration of 1 mg/mL during 1 h at 37 °C in 5% CO_2_. Reaction was stopped adding 150 µL of NaOH 2N. *A* in each well represents acid phosphatase activity from all living cells and was read in an automated plate spectrophotometer at 405 nm. *A* obtained in wells containing HGEC without platelets was subtracted from that obtained in wells with HGEC and platelets. The percentage of adhered platelets was calculated considering that *A* from wells without toxin treatment represents 100%.

### 4.9. Patients and Samples

The study was approved by the Hospital Ethical Committees: the Comité de Bioética del Hospital Municipal del Niño de San Justo, San Justo, Provincia de Buenos Aires, Argentina and the Comité de Bioética del Hospital General Jose Penna, Bahia Blanca, Provincia de Buenos Aires, Argentina. All of the patients were enrolled after informed consent was obtained from their parents. The study included 23 children during the acute period of HUS. All of the patients developed HUS after a prodrome of gastroenteritis with bloody diarrhea. There were 8 girls and 15 boys in the study. Clinical and biochemical data of patients are presented in Table 1. Blood samples (2 mL) were obtained by venopuncture into EDTA plastic tubes, before dialysis and/or transfusion at different days after the onset of diarrhea (Table 1). Blood samples from healthy controls (HC) were collected and processed identically. Plasma was obtained by blood centrifugation at RT at 800× *g* for 10 min, aliquoted and stored at −80 °C until analysis.

### 4.10. sCD40L Measurement

sCD40L levels in plasma and supernatants were determined by using an ELISA kit following manufacturer’s instructions (e-Biosciences, San Diego, CA, USA). Assay sensitivity was 10 to 0.16 ng/mL.

### 4.11. Plasma Depletion of sCD40L

This method was adapted from Zhang et al. [72]. 96-well plate was coated with 2 µg/well anti-CD40L (Becton Dickinson, Franklin Lakes, NJ, USA) overnight at 4 °C. Wells were washed three times with PBS, blocked with PBS-BSA (0.5%) during 1h. They were then washed three times with PBS and incubated with 100 µL of plasma from HUS or HC at RT for 2 h. Then, depleted plasmas were collected and stored at −80 °C until analysis. Non-depleted plasmas were treated in identical conditions but without anti-CD40L coating. Those plasmas from HUS patients with sCD40L levels higher than 3 ng/mL were selected for depletion and the effectiveness of the procedure was confirmed by ELISA.

### 4.12. PBMC Isolation

Blood from healthy donors was diluted 1:2 with saline, layered on a Ficoll-Hypaque cushion (Ficoll Pharmacia, Uppsala, Sweden; Hypaque, Winthrop Products, Buenos Aires, Argentina) and centrifuged at 400× *g* for 30 min, as previously described [73]. Peripheral blood mononuclear cells (PBMC) were collected, washed twice, and suspended in RPMI. Viability of PBMC was more than 96% as determined by trypan blue exclusion test.

### 4.13. ROS Generation Measurement

PBMC (1 × 10^6^) were incubated 1 h with 10% of HUS and HC plasma, depleted or not for sCD40L, at 37 °C in 5% CO_2_, then washed in PBS and resuspended in 200 µL of RPMI. DHR-123 (5 μM) was added for 15 min at 37 °C. Afterwards, the cells were washed and suspended in 200 μL of Isoflow (International Link, SA, Buenos Aires, Argentina). Green fluorescence was measured on 10,000 events with a Becton Dickinson (Franklin Lakes, NJ, USA) fluorescence activated cell sorter (FACScan) and analysed using the Cell-Quest program. Monocytes were identified and gated using forward/side-scatter (FSC/SSC) dot-plot profiles and CD14 staining by using anti-CD14 (Becton Dickinson, Franklin Lakes, NJ, USA).

### 4.14. Data Analysis

Data are presented as median and interquartile range. All data were analyzed by a non-parametric Kruskal-Wallis test followed by the Dunnett’s multiple comparisons test. The non-parametric Spearman test was used for correlations. A *p* value lower than 0.05 was considered to be statistically significant.

## Figures and Tables

**Table 1:** Clinical and biochemical data of patients with HUS. According to Gianantonio et al’s criteria, 23 patients were retrospectively classified as mild-moderate cases (grade 1–2: less than 7 days of anuria) or severe cases (grade 3: more than 7 days of anuria). Data are presented as median (interquartile range).

**Severity of Renal Dysfunction**		
**General Parameters**	**Grade 1 y 2** (*n* = 13)	**Grade 3** (*n* = 10)
Age (month)	45.0 (22.0–81.2)	21.5 (10.5–60.5)
Time from the onset of diarrhea (days) ^a^	4.0 (3.0–5.0)	4.5(1.7–9.7)
**Blood and Renal Parameters**		
Platelets (×10^9^/L)	77.5 (39.2–34.5)	45.0 (34.5–57.0)
Leukocytes (×10^9^/L)	14.0 (8.9–22.3)	17.4 (13.8–31.0)
Hematocrit (%)	24.0 (19.3–26.0)	24.2 (23.0–25.7)
Urea (mmol/L)	15.6 (10.5–25.9)	39.7 (26.6–54.3)
Creatinine (µmol/L)	110.5 (61.8–223.2)	419.9 (235.4–795.6)

^a^ The number of days between the onset of diarrhea and time of blood sample collection.
